# Surgery of the Lungs and Pleura

**Published:** 1894-09-15

**Authors:** 


### SURGERY OF THE LUNGS AND PLEURA.

**Injuries of the Lung.**—Lopez[7] relates a case of hernia of the lung complicating an incised wound of the chest. Reduction was impossible, and symptoms of gangrene of the lung were imminent, so it was decided to resect the protruding portion of lung. It was ligatured in two parts, cut off, and the pedicle returned into the pleural cavity. Some pyo-thorax resulted, but eventually the wound healed without adhesion of the lung to the chest wall, and the patient recovered by the twenty-second day.

An exceedingly interesting case of traumatic rupture of the lung without fracture of the ribs is reported by Dr. Comte.[8] A man was struck on the front of the right side of the chest by the beam of a machine. Urgent dyspnœa and profuse hæmoptysis resulted, and there were physical signs of consolidation of the upper lobe of the lung. The patient lived a week. At the autopsy an extensive rent in the lung was discovered at the junction of the upper third with the lower two-thirds, with consolidation around. The tear was situated on the external border of the lung where the old adhesions existed. Dr. Comte was of opinion that the lung ruptured because it was firmly held by the adhesions, and thus not able to recoil from the blow, but we can hardly suppose that without any adhesion the lung would retreat from the chest wall unless at the same time air entered the pleural cavity, and we think the explanation usually given, that at the moment of the accident the patient took a deep inspiration and then closed the glottis, so that the full force of the blow would come on the expanded lung, was more probably the correct one.

**Diagnosis and Treatment of Abscess of the Lung.**—A case is reported by Mr. Morton, in the *Lancet*,[9] in which general bronchiectasis of one lung, the result of the impaction of a foreign body in the right bronchus, gave rise to physical signs, suggestive of a large cavity rather than several small ones scattered throughout the lung. A similar case, under the care of Dr. Coupland and Mr. Pearce Gould, is referred to. In Mr. Morton's case, the patient, a girl nine years of age, had a metal cap impacted in her bronchus for three years. Ever since the accident there had been persistent cough, and in August last she began for the first time to expectorate pus in large quantities. The metal cap was never found in the expectoration. At times the latter was very offensive. She came under Mr. Morton's care in November, 1893. The right side of the chest was shrunken and flattened and hardly moved with inspiration. The heart was beating just inside the right nipple. The right side of the chest below the nipple level in front, and in the axilla, and below the angle of the scapula behind was absolutely dull, and above this level the resonance was much impaired. Over the higher more resonant area the breath sounds were cavernous; intensely so just between the scapula and the spine at the level of the spine of the scapula, and only a little less so over the outer part of the right apex and in the upper axilla. Pectoriloquy was most intense where the breath sounds were most cavernous. An exploring syringe withdrew pus in the upper axilla. An attempt was made to open into what was supposed to be a large cavity here, and the child died from anæsthesia, the bronchial tubes becoming filled with pus, which she was unable to expectorate in the anæsthetic condition. At the post-mortem examination the lung was found sclerosed and greatly shrunken, with the bronchial tubes all through the organ greatly dilated, forming a series of small abscesses, but the largest did not exceed the size of a walnut. The heart was so much drawn over that the great vessels were close to the chest wall at the right apex. Mr. Morton considered that the physical signs of a large cavity, were due to conduction through the fibroid lung around the larger branches of the bronchial tubes. With regard to the operation it is important to observe that after excising a portion of rib, and stitching the lung to the parietal pleura, on thrusting a trochar and cannula into the lung, instead of penetrating that organ, it pushed it before it and tore away the sutures and adhesions existing at the spot, as shown by the post-mortem examination.

[7] Siglo, Medico, April, 1894; and Brit. Med. Journ., April 28, p. 65.
[8] Journal des Praticiens, Feb. 21, 1894; and Lancet, March 17, p. 693.
[9] Lancet, June 9, 1894, p. 1439.

